# The Association of Retinal age gap with metabolic syndrome and inflammation

**DOI:** 10.1111/1753-0407.13364

**Published:** 2023-03-14

**Authors:** Zhuoting Zhu, Dan Liu, Ruiye Chen, Wenyi Hu, Huan Liao, Katerina Kiburg, Jason Ha, Shuang He, Xianwen Shang, Yu Huang, Wei Wang, Honghua Yu, Xiaohong Yang, Mingguang He

**Affiliations:** ^1^ Department of Ophthalmology Guangdong Provincial People's Hospital (Guangdong Academy of Medical Sciences), Southern Medical University Guangzhou China; ^2^ Centre for Eye Research Australia; Ophthalmology University of Melbourne Melbourne Australia; ^3^ Ophthalmology, Department of Surgery University of Melbourne Melbourne Australia; ^4^ Population Health Sciences German Centre for Neurodegenerative Diseases (DZNE) Bonn Germany; ^5^ State Key Laboratory of Ophthalmology, Zhongshan Ophthalmic Center Sun Yat‐sen University Guangzhou China

**Keywords:** inflammation, metabolic syndrome, retinal age gap, 视网膜年龄差, 代谢综合征, 炎症

## Abstract

**Background:**

Metabolic syndrome (MetS) is a clustering of cardiometabolic components, posing tremendous burdens in the aging society. Retinal age gap has been proposed as a robust biomarker associated with mortality and Parkinson's disease. Although MetS and chronic inflammation could accelerate the aging process and increase the risk of mortality, the association of the retinal age gap with MetS and inflammation has not been examined yet.

**Methods:**

Retinal age gap (retina‐predicted age minus chronological age) was calculated using a deep learning model. MetS was defined as the presence of three or more of the following: central obesity, hypertension, dyslipidemia, hypertriglyceridemia, and hyperglycemia. Inflammation index was defined as a high‐sensitivity C‐reactive protein level above 3.0 mg/L. Logistic regression models were used to examine the associations of retinal age gaps with MetS and inflammation.

**Results:**

We found that retinal age gap was significantly associated with MetS and inflammation. Specifically, compared to participants with retinal age gaps in the lowest quartile, the risk of MetS was significantly increased by 10% and 14% for participants with retinal age gaps in the third and fourth quartile (odds ratio [OR]:1.10; 95% confidence interval [CI], 1.01,1.21;, *p* = .030; OR: 1.14, 95% CI, 1.03,1.26; *p* = .012, respectively). Similar trends were identified for the risk of inflammation and combined MetS and inflammation.

**Conclusion:**

We found that retinal age gaps were significantly associated with MetS as well as inflammation. Given the noninvasive and cost‐effective nature and the efficacy of the retinal age gap, it has great potential to be used as a screening tool for MetS in large populations.

## Graphical abstract

**Highlights**

- Retinal age gap is a non‐invasive and cost‐effective aging biomarker.
- Retinal age gap was significantly associated with metabolic syndrome and inflammation.
- Retinal age gap could be potentially used as a screening tool for metabolic syndrome in large populations.

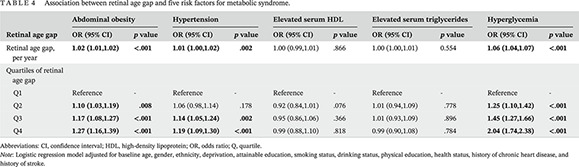

## 摘要

**背景:** 代谢综合征(MetS)是心脑代谢成分的聚集, 在老龄化社会构成巨大的负担。视网膜年龄差被认为是与死亡率和帕金森病相关的一种强有力的生物标志物。虽然MetS和慢性炎症可以加速衰老过程并增加死亡风险, 但视网膜年龄差与MetS和炎症之间的关系尚未被研究。

**方法:** 使用深度学习模型计算视网膜年龄差(视网膜预测年龄减去实际年龄)。将MetS定义为以下三项或以上存在:中心性肥胖、高血压、血脂异常、高三酰甘油血症和高血糖。炎症指数定义为高敏C‐反应蛋白(CRP)水平大于3.0 mg/L。使用Logistic回归模型考察视网膜年龄差与MetS和炎症的关联。

**结果:** 我们发现视网膜年龄差与MetS和炎症显著相关。具体而言, 与视网膜年龄差在最低四分位数的参与者相比, 视网膜年龄差在第三和第四分位数的参与者的MetS风险分别增加了10%和14%(OR [95%CI]:1.10(1.01, 1.21), P = 0.030; OR [95%CI]:1.14(1.03, 1.26), P = 0.012)。在炎症合并MetS和炎症的风险方面也发现了类似的趋势

结论:视网膜年龄差与MetS以及炎症显著相关。鉴于其非侵入性和低成本的特点, 以及其有效性, 视网膜年龄差有巨大的潜力作为大规模人群的MetS筛查工具。

## 1 INTRODUCTION

Metabolic syndrome (MetS) is a clustering of cardiometabolic components, including abdominal obesity, hypertension, elevated glucose levels, as well as dyslipidemia, which are highly prognostic of type 2 diabetes mellitus and cardiovascular diseases (CVD), causing morbidity and mortality eventually.^1^, ^2^ Notably, inflammation has been reported to play a crucial role in the pathology of MetS.^3^, ^4^ The incidence of MetS rises exponentially as the population ages, posing a tremendous burden to individuals and families in the aging society.^5^

Early detection and risk stratification of MetS are needed to improve prevention and early intervention strategies of the diabetes and CVDs. A growing number of studies have investigated several potential screening tools for the MetS including anthropometric measurements and blood tests.^6^, ^7^ Among all the tools proposed, noninvasive measurements such as body mass index, waist circumference, and blood pressure stand out.^8^, ^9^ However, measurement errors and ethnicity heterogeneity have limited their further application for screening in large populations.^10^, ^11^, ^12^ Therefore, a novel, reliable, and noninvasive method is urgently warranted to accurately screening for MetS.

Retinal age has been recently proposed as a novel reliable aging biomarker. Our research group leveraged deep learning (DL) to accurately predict chronological age based on retinal fundus images in healthy populations.^13^ Retinal age gap was defined as the difference between retina‐predicted age and chronological age, showing the deviations from normal aging. Our group has verified that the retinal age gap could independently predict future death events and age‐related diseases events including cardiovascular diseases and Parkinson's disease.^13^, ^14^, ^15^ Taken together, retinal age could reflect the overall health and thus has been considered as a reliable indicator of aging. As age is an independent risk factor for MetS, we hypothesized that retinal age gaps may be associated with MetS.

Herein, we aimed to investigate the associations of retinal age gap with MetS in a large population based on UK Biobank study. In addition, the association between retinal age gap and inflammation as a pathogenic component of MetS was also investigated in the present study.

## 2 METHODS

### 2.1 Study population

The UK Biobank is a population‐based cohort study with over 500 000 participants recruited in the United Kingdom between 2006 and 2010. A total of 22 assessment centers were set across England, Wales, and Scotland. Approximately 9.2 million residents aged 40–69 years within 25 miles of a nearest assessment center were invited to participant in this study, among whom 5.5% were enrolled in the baseline assessment. All participants completed comprehensive health care questionnaires, detailed physical measurements, and biological sample collections. The overall study protocols and data are available elsewhere.^16^ This study was restricted to a subset of participants with metabolic syndrome and/or inflammation data available at the initial assessment (March 2006 to December 2010).

### 2.2 Ethical approval

The UK Biobank Study received approval from the National Information Governance Board for Health and Social Care and the National Health Service North West Multicentre Research Ethics Committee (11/NW/0382). Written informed consent was obtained from all participants in accordance with the Declaration of Helsinki.

### 2.3 Fundus photography

Ophthalmic examinations were introduced from 2010. LogMAR visual acuity, autorefraction, and keratometry (Tomey RC5000, Tomey GmbH, Nuremberg, Germany); intraocular pressure (Ocular Response Analyzer, Reichert, New York, USA); and paired retinal fundus and optical coherence tomography imaging (OCT, Topcon 3D OCT 1000 Mk2, Topcon Corp, Tokyo, Japan) examinations were performed. A 45‐degree nonmydriatic and nonstereo fundus image centered on the macula with the optic disc included was taken for each eye. A total of 131 238 images from 66 500 participants were obtained from the UK Biobank study. A total of 80 170 images from 46 970 participants who passed the image quality check were used for the following analyses.

### 2.4 Estimation of retina age and retinal age gap

Retinal age was predicted according to the methods described by Zhu et al.^13^ Briefly, only color fundus images were fed into a DL model using an Xception architecture to predict biological age. DL is the advanced subset of machine learning with multiple neural networks and could self‐learn complex representations without requiring human engineering and domain expertise to design feature extractors or calculation methods.

The difference between the retinal age predicted by the DL model and chronological age was defined as the retinal age gap. A positive retinal age gap indicated an “older” appearing retina, whereas a negative one indicated a “younger” appearing retina.

### 2.5 Metabolic syndrome and inflammation index

MetS was defined as the presence of three or more of the following: central obesity, hypertension, dyslipidemia, hypertriglyceridemia, and hyperglycemia.^1^, ^17^ Waist circumference was measured at the smallest part of the trunk using a 200‐cm tape measure (SECA). Central obesity was defined as a waist circumference of 88 cm or above for women and 102 cm or above for men. Systolic and diastolic blood pressure were measured twice using an IntelliSense blood pressure monitor model HEM‐907XL (Omron) after participants rested for at least 5 min and the averages were taken as the final results.^18^ Hypertension was defined as a systolic blood pressure of ≥130 mm Hg and/or a diastolic blood pressure of ≥80 mm Hg or taking antihypertensive drugs. Dyslipidemia was defined as levels of high‐density lipoprotein (HDL) cholesterol <50 mg/dL in women and <40 mg/dL in men. A triglyceride level ≥150 mg/dL was defined as hypertriglyceridemia. Hyperglycemia was defined as fasting blood glucose levels >110 mg/dL or taking hypoglycemic medication or using insulin. Inflammation index was defined as a high‐sensitivity C‐reactive protein (CRP) level >3.0 mg/L.

### 2.6 Demographic and health variables

Demographic factors and health variables included baseline age, sex, ethnicity (recorded as white and nonwhite), Townsend deprivation indices (an area‐based proxy measure for socioeconomic status), education attainment (recorded as college or university degree, and others), smoking status (recorded as current/previous and never), drinking status (recorded as current/previous and never), physical activity level (recorded as above moderate/vigorous/walking recommendation and not), history of heart disease (angina or heart attack), history of stroke, and general health status (recorded as excellent/good and fair/poor).

### 2.7 Statistical analyses

Data are presented as mean (SD) or median (interquartile range, IQR) for continuous variables and numbers (percentages) for categorical variables. The differences of the baseline characteristics of the study population among retinal age gap quartiles were compared using analysis of variance and chi‐square test. The differences of the baseline characteristics between the MetS group and non‐MetS group were compared using chi‐square test and/or *t* test. Logistic regression models were applied to investigate the association between retinal age gap (independent variable) and MetS and inflammation (outcome), respectively. We then investigated associations between retinal age gaps at different quartiles with metabolic syndrome and inflammation. The associations between retina age gap and abdominal obesity, hypertension, elevated serum HDL, elevated serum triglycerides, and hyperglycemia were also examined respectively by logistic regression models. All logistic regression models were adjusted for baseline age, sex, ethnicity, Townsend deprivation indices, educational level, smoking status, drinking status, physical activity level, general health status, history of heart disease, and history of stroke. Odds ratios (OR) with their 95% confidence intervals (CIs) were reported. Variance inflation factors (VIF) procedure was used to test collinearity for all variables and all covariables' VIF were <2. For all the analyses, complete data were used. A two‐sided *p* value of <.05 indicated statistical significance. Analyses were performed using Stata (version 13, StataCorp, Texas, USA).

## 3 RESULTS

### 3.1 Study sample

Of 35 918 included participants, the mean (SD) age was 56.6 (8.04) years, and 20 002 (55.7%) were women. Table 1 shows baseline characteristics of the study participants overall and stratified by quartiles of retinal age gaps. As shown, participants with a higher quartile of retinal age gaps were more likely to be younger, of female gender, of nonwhite ethnicity, nonsmokers, physical inactivity, less healthy in general status, more deprived, better educated, and without a history of chronic heart diseases and stroke (*p* < .001).

**TABLE 1:** Baseline characteristics of the study participants stratified by quartiles of retinal age gap.

Baseline characteristics	Total	Retinal age gap				Test results	*p* value
		Q1	Q2	Q3	Q4		
*N*	35 918	8980	8979	8980	8979		‐
Age, years, mean (SD)	56.6 (8.04)	63.1 (4.80)	59.3 (6.43)	54.7 (7.34)	49.9 (6.43)	7424.45^a^	**<.001**
Sex, *n* (%)							
Men	15 916 (44.3)	4409 (49.1)	3970 (44.2)	3812 (42.4)	3725 (41.5)	125.08^b^	**<.001**
Women	20 002 (55.7)	4571 (50.9)	5009 (55.8)	5168 (57.6)	5254 (58.5)		
Ethnicity, *n* (%)							
White	33 480 (93.2)	8475 (94.4)	8431 (93.9)	8318 (92.6)	8256 (92.0)	53.42^b^	**<.001**
Nonwhite	2438 (6.79)	505 (5.62)	548 (6.10)	662 (7.37)	723 (8.05)		
Townsend index, mean (SD)	−1.09 (2.96)	−1.45 (2.79)	−1.22 (2.88)	−0.99 (3.02)	−0.69 (3.08)	108.07^a^	**<.001**
Attainable education, *n* (%)							
Above college/university	12 462 (34.7)	2723 (30.3)	2976 (33.1)	3186 (35.5)	3577 (39.8)	192.52^b^	**<.001**
Below college/university	23 456 (65.3)	6257 (69.7)	6003 (66.9)	5794 (64.5)	5402 (60.2)		
Smoking status, *n* (%)							
Never	19 793 (55.4)	4869 (54.5)	4857 (54.3)	4883 (54.6)	5184 (58.0)	33.80^b^	**<.001**
Former/current	15 945 (44.6)	4058 (45.5)	4081 (45.7)	4056 (45.4)	3750 (42.0)		
Drinking status, *n* (%)							
Never	1586 (4.43)	443 (4.94)	357 (3.98)	396 (4.42)	390 (4.36)	9.94^b^	**.019**
Former/current	34 223 (95.6)	8516 (95.1)	8607 (96.0)	8554 (95.6)	8546 (95.6)		
Meeting physical education recommendation, *n* (%)							
No	5307 (18.1)	1132 (15.7)	1274 (17.4)	1383 (18.8)	1518 (20.2)	56.87^b^	**<.001**
Yes	24 084 (81.9)	6096 (84.3)	6039 (82.6)	5962 (81.2)	5987 (79.8)		
Health status, *n* (%)							
Excellent/good	24 823 (69.5)	6466 (72.3)	6304 (70.5)	6152 (69.0)	5901 (66.3)	81.97^b^	**<.001**
Fair/poor	10 895 (30.5)	2477 (27.7)	2644 (29.5)	2771 (31.0)	3003 (33.7)		
History of chronic heart diseases, *n* (%)							
No	34 383 (95.7)	8399 (93.5)	8547 (95.2)	8667 (96.5)	8770 (97.7)	208.98^b^	**<.001**
Yes	1535 (4.27)	581 (6.47)	432 (4.81)	313 (3.49)	209 (2.33)		
History of stroke, *n* (%)							
No	35 375 (98.5)	8793 (97.9)	8834 (98.4)	8865 (98.7)	8883 (98.9)	35.32^b^	**<.001**
Yes	543 (1.51)	187 (2.08)	145 (1.61)	115 (1.28)	96 (1.07)		

*Note*: The bold values showed that the *p* value is less than 0.05, which indicated statistical significance in the current analysis.

Abbreviation: Q, quartile.

^a^ is the *F* value of analysis of variance.

^b^ is the degree of freedom of chi square.

**FIGURE 1:**
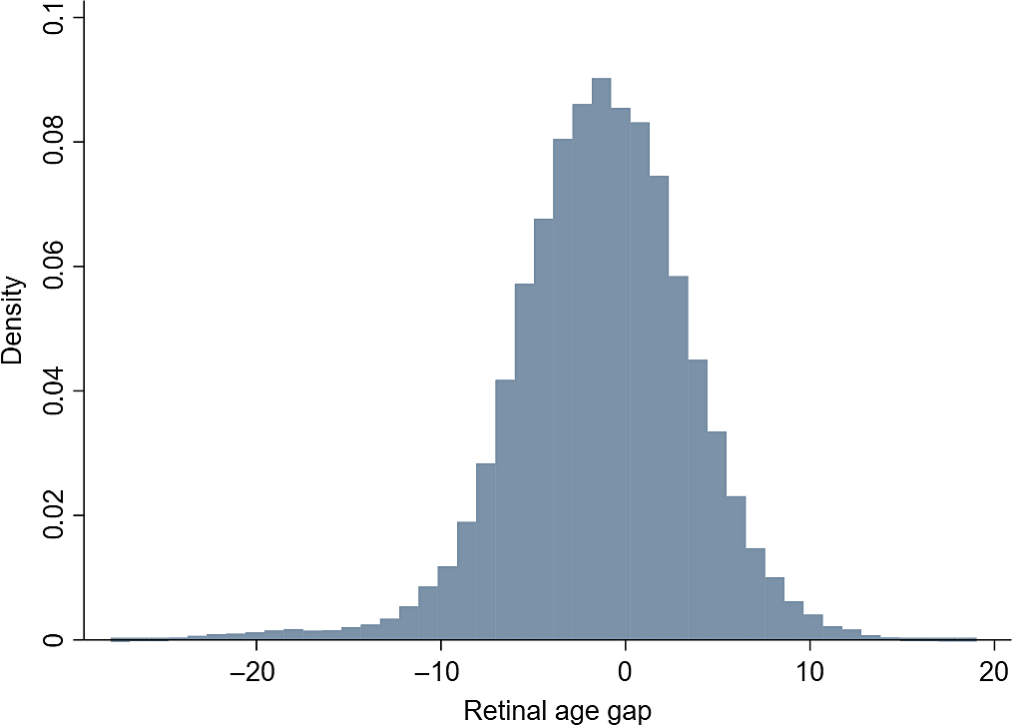
The distribution of retinal age gap of the included participants.

The distribution of retinal age gap of the included participants is shown in Figure 1. The mean (SD) and median (IQR) of the retinal age gap were −1.31 (4.82) and −1.18 (−4.18 to 1.79). The retinal age gap was divided into four groups of equal size, with Q1 as the lowest 25% retinal age gap, Q2 as the lowest 25%–50%, Q3 as 50%–75%, and Q4 as the highest 25% of retinal age gaps. The retinal age gap ranges for the four quartiles are Q1 (−27.9 to −4.18), Q2 (−4.18 to −1.18), Q3 (−1.18 to 1.79), and Q4 (1.79 to 19.0). We also added the fundus photographs of Q1‐Q4 stages in the Figure S1. Compared to the fundus images in Q1, the fundus images in Q4 tended to have more retinal vascular changes including arteriolar narrowing and blood vessel tortuosity.

### 3.2 Metabolic syndrome and inflammation

Table 2 presents baseline characteristics of the study participants stratified by MetS. Compared to the nonmetabolic syndrome group, participants with MetS tend to be older, of male gender, former/current smokers, nondrinkers, physical inactivity and with greater deprivation, poor education, and poor general health status, and with a history of chronic heart diseases and stroke (*p* < 0.001).

**TABLE 2:** Baseline characteristics of the study participants stratified by metabolic syndrome.

Baseline characteristics	Metabolic syndrome group	Nonmetabolic syndrome group	Test result	*p* value
*N*	7921	27 997		‐
Age, years, mean (SD)	57.9 (0.09)	56.4 (0.05)	−14.25^a^	**<.001**
Sex, *n* (%)				
Men	3837 (48.4)	12 079 (43.1)	70.20^b^	**<.001**
Women	4084 (51.6)	15 918 (56.9)		
Ethnicity, *n* (%)				
White	7371 (93.1)	26 109 (93.3)	0.39^b^	.532
Nonwhite	550 (6.94)	1888 (6.74)		
Townsend index, mean (SD)	−0.88 (0.03)	−1.15 (0.02)	−7.19^a^	**<.001**
Attainable education, *n* (%)				
Above college/university	2127 (26.9)	10 335 (36.9)	275.89^b^	**<.001**
Below college/university	5794 (73.2)	17 662 (63.1)		
Smoking status, *n* (%)				
Never	4120 (52.4)	15 673 (56.2)	36.22^b^	**<.001**
Former/current	3742 (47.6)	12 203 (43.8)		
Drinking status, *n* (%)				
Never	506 (6.42)	1080 (3.87)	94.49^b^	**<.001**
Former/current	7378 (93.6)	26 845 (96.1)		
Meeting physical education recommendation, *n* (%)				
No	1475 (23.5)	3832 (16.6)	162.58^b^	**<.001**
Yes	4787 (76.5)	19 297 (83.4)		
Health status, *n* (%)				
Excellent/good	4340 (55.2)	20 483 (73.5)	976.22^b^	**<.001**
Fair/poor	3256 (44.8)	7369 (26.5)		
History of chronic heart diseases, *n* (%)				
No	7377 (93.1)	27 006 (96.5)	167.17^b^	**<.001**
Yes	544 (6.87)	991 (3.54)		
History of stroke, *n* (%)				
No	7762 (98.0)	27 613 (98.6)	16.76^b^	**<.001**
Yes	159 (2.01)	384 (1.37)		

*Note*: The bold values showed that the *p* value is less than 0.05, which indicated statistical significance in the current analysis.

Abbreviation: Q, quartile.

^a^ is the *t* value of *t* test.

^b^ is the degree of freedom of chi square.

### 3.3 Retinal age gap and metabolic syndrome and/or inflammation

Associations of retinal age gap with metabolic syndrome and/or inflammation are reported in Table 3. Each year increase in the retinal age gap was associated with a 1% risk increase of MetS (OR: 1.01; 95% CI, 1.00,1.02; *p* = .016), a 1% risk increase of inflammation (OR: 1.01; 95% CI, 1.00,1.02; *p* = .021), and a 1% risk increase of MetS and inflammation combined (OR: 1.01; 95% CI, 1.00,1.02; *p* = .011) in the fully adjusted model. Compared to participants with the lowest quartile of retinal age gaps, the risk of MetS significantly increased by 10% and 14% respectively for participants with retinal age gap in the third and fourth quartiles (OR: 1.10; 95% CI, 1.01,1.21; *p* = .030; OR: 1.14; 95% CI, 1.03,1.26; *p* = .012, respectively). A similar trend was identified for the risk of inflammation that participants with retinal age gaps in the third and fourth quartiles had 10% and 25% increased risks (OR: 1.10; 95% CI, 1.01,1.21; *p* = .048; OR: 1.25; 95% CI, 1.12,1.38; *p* < .001) compared to those in the first quartile, respectively. A significant association existed between retinal age gaps and a combined condition of inflammation and MetS. Table 4 shows the association between retinal age gaps and the five specific subcomponents of MetS. Per year increase in retinal age gaps was associated with a 2% risk increase in abdominal obesity (OR: 1.02, 95% CI, 1.01,1.02; *p* < .001), a 1% risk increase in hypertension (OR: 1.01; 95% CI, 1.00,1.02; *p* = .002), a 6% risk increase in hyperglycemia (OR: 1.06, 95% CI, 1.04,1.07; *p* < .001) but not associated with elevated serum HDL and elevated serum triglycerides. Compared with the participants with the lowest retinal age gap quartile, participants with higher quartiles tend to have a 10% to 27% risk increase in abdominal obesity and 14% to 19% risk increase in hypertension. Moreover, participants with the higher quartiles of retinal age gaps were associated with a 25% to 104% risk increase in hyperglycemia (OR: 1.25; 95% CI, 1.10,1.42; *p* < .001; OR: 1.45; 95% CI, 1.27,1.66;, *p* < .001; OR: 2.04; 95% CI, 1.74,2.38; *p* < .001, respectively).

**TABLE 3:** Association of retinal age gap with metabolic syndrome and/or inflammation.

Retinal age gap	Metabolic syndrome		Inflammation		Combined	
	OR (95% CI)	*p* value	OR (95% CI)	*p* value	OR (95% CI)	*p* value
Retinal age gap, per year	**1.01 (1.00,1.02)**	**.016**	**1.01 (1.00,1.02)**	**.021**	**1.01 (1.00,1.02)**	**.001**
Quartiles of retinal age gap						
Q1	Reference	‐	Reference	‐	Reference	‐
Q2	1.05 (0.96,1.14)	.283	1.02 (0.94,1.12)	.614	1.04 (0.964,1.11)	.347
Q3	**1.10 (1.01,1.21)**	**.030**	**1.10 (1.00,1.21)**	**.048**	**1.11 (1.03,1.20)**	**.007**
Q4	**1.14 (1.03,1.26)**	**.012**	**1.25 (1.12,1.38)**	**<.001**	**1.20 (1.10,1.31)**	**<.001**

*Note*: The bold values showed that the *p* value is less than 0.05, which indicated statistical significance in the current analysis.

Abbreviations: CI, confidence interval; OR, odds ratio; Q, quartiles.

*Note*: Logistic regression model adjusted for baseline age, sex, ethnicity, deprivation, attainable education, smoking status, drinking status, physical education, health status, history of chronic heart disease, and history of stroke.

**TABLE 4:** Association between retinal age gap and five risk factors for metabolic syndrome.

Retinal age gap	Abdominal obesity		Hypertension		Elevated serum HDL		Elevated serum triglycerides		Hyperglycemia	
	OR (95% CI)	*p* value	OR (95% CI)	*p* value	OR (95% CI)	*p* value	OR (95% CI)	*p* value	OR (95% CI)	*p* value
Retinal age gap, per year	**1.02 (1.01,1.02)**	**<.001**	**1.01 (1.00,1.02)**	**.002**	1.00 (0.99,1.01)	.866	1.00 (1.00,1.01)	0.554	**1.06 (1.04,1.07)**	**<.001**
Quartiles of retinal age gap										
Q1	Reference	‐	Reference	‐	Reference	‐	Reference	‐	Reference	‐
Q2	**1.10 (1.03,1.19)**	**.008**	1.06 (0.98,1.14)	.178	0.92 (0.84,1.01)	.076	1.01 (0.94,1.09)	.778	**1.25 (1.10,1.42)**	**<.001**
Q3	**1.17 (1.08,1.27)**	**<.001**	**1.14 (1.05,1.24)**	**.002**	0.95 (0.86,1.06)	.366	1.01 (0.93,1.09)	.896	**1.45 (1.27,1.66)**	**<.001**
Q4	**1.27 (1.16,1.39)**	**<.001**	**1.19 (1.09,1.30)**	**<.001**	0.99 (0.88,1.10)	.818	0.99 (0.90,1.08)	.784	**2.04 (1.74,2.38)**	**<.001**

*Note*: The bold values showed that the *p* value is less than 0.05, which indicated statistical significance in the current analysis.

Abbreviations: CI, confidence interval; HDL, high‐density lipoprotein; OR, odds ratio; Q, quartile.

*Note*: Logistic regression model adjusted for baseline age, gender, ethnicity, deprivation, attainable education, smoking status, drinking status, physical education, health status, history of chronic heart disease, and history of stroke.

## 4 DISCUSSION

In a large population of middle‐aged and older adults, we found that the retinal age gap was significantly associated with MetS and inflammation. Specifically, compared to participants with retinal age gaps in the lowest quartile, the risk of MetS was significantly increased by 10% and 14% respectively for participants with retinal age gaps in the third and fourth quartile. Similar trends were identified for the risk of inflammation and combined MetS and inflammation.

Previous studies have provided valuable clues for our investigations about the associations of retinal age gaps with MetS and inflammation. First, structural defects of the retina were found in patients with MetS and inflammation.^19^ For example, the patients with MetS and high levels of CRP had thinner inner retinal layers and photoreceptor layer in OCT segmentation analysis.^20^, ^21^ Second, retinal microvascular signs were identified in the fundus images from MetS patients. A population‐based study suggested that MetS was associated with retinal microvascular signs (microaneurysms, retinal hemorrhages, arteriovenous nicking, and focal arteriolar narrowing) based on retinal photographs.^22^ Moreover, MetS and its components, as well as CRP, a marker of inflammation, have been associated with retinal diseases including age‐related macular degeneration,^23^, ^24^ retinopathy,^25^, ^26^ and glaucoma,^27^ resulting in significant increases in morbidity and mortality.^2^, ^23^

Ours findings might be explained by several underlying mechanisms. MetS and chronic inflammation may contribute to the retinal age gap between biological age and chorological age, as both of the conditions accelerate aging process and increase the risk of age‐related diseases.^23^ Moreover, early signs of MetS could be presented in the retina at an early stage. The microvascular and macrovascular dysfunctions in MetS are the fundamental pathology in the development of MetS.^28^ The retina, as a highly vascular organ, could serve as an instant window to the systemic vasculature, having the potential to catch the early vascular changes of MetS. Of note, the attention maps of our DL model for the prediction of retina age exactly highlighted areas near the retinal vessels.

The retinal age gap has provided a novel and reliable screening method for MetS and inflammation. Compared with previous screening tools based on blood tests or anthropometric measurements, the retinal age gap is measured by a deep learning model to integrate all features from fundus images automatically, so that it could minimize the manual assessments error and avoid invasive testing. Therefore, it has great potentials to be used as a diagnosis biomarker, characterized by noninvasiveness, reliability, and objectiveness. This guarantees its further application in screening for MetS as well as inflammation in large populations. Moreover, an early detection and risk stratification of MetS and inflammation could help to promote the prevention and intervention strategies for chronic diseases such as diabetes and CVDs, which may effectively alleviate the economic burden of the whole society.

Although the large population, standardized protocol, high quality of fundus image data, and objective measures based on a deep learning model have enhanced the robustness of our findings, the current study harbors several limitations. First, causality could not be drawn from this cross‐sectional study. Second, our results might underestimate the effects of retinal age gap on MetS, as individuals with poor health would be less likely to participate in this study. Longitudinal data are needed to investigate the association between retinal age gaps and incident MetS.

## 5 CONCLUSION

We found that retinal age gaps were significantly associated with MetS as well as inflammation. Based on a DL model, the retinal age gap has great potentials to be used as a noninvasive, reliable, and objective tool for the screening of MetS in large populations. Longitudinal data are further needed to confirm the association between retinal age gaps and incident MetS.

## AUTHOR CONTRIBUTIONS

Study concept and design: Zhuoting Zhu, Dan Liu, Ruiye Chen, Mingguang He. Acquisition, analyses, or interpretation: all authors. Drafting of the manuscript: Ruiye Chen, Zhuoting Zhu, Dan Liu. Critical revision of the manuscript for important intellectual content: Wenyi Hu, Katerina Kiburg, Jason Ha, Wei Wang, Honghua Yu, Xiaohong Yang, Mingguang He. Statistical analyses: Ruiye Chen, Zhuoting Zhu, Dan Liu, Xianwen Shang. Obtained funding: Zhuoting Zhu, Wei Wang, Honghua Yu, Xiaohong Yang, Mingguang He. Administrative, technical, or material support: Wei Wang, Honghua Yu, Xiaohong Yang, Mingguang He. Study supervision: Wei Wang, Honghua Yu, Xiaohong Yang, Mingguang He.

## CONFLICT OF INTEREST

We declare no competing interests.

## Supporting information


**Figure S1.** Examples of fundus photographs in different quartiles (Q1–Q4) of retinal age gap in participants aged 50 years.
