# Supplementary material for: The Association of Retinal age gap with metabolic syndrome and inflammation
Source: J Diabetes. 2023 Mar 14;15(3):237–45. doi: 10.1111/1753-0407.13364 (PMC10036256; doi:10.1111/1753-0407.13364)

Supplementary Figure 1. Examples of fundus photographs in different quartiles (Q1-Q4) of retinal age gap in participants aged 50 years.


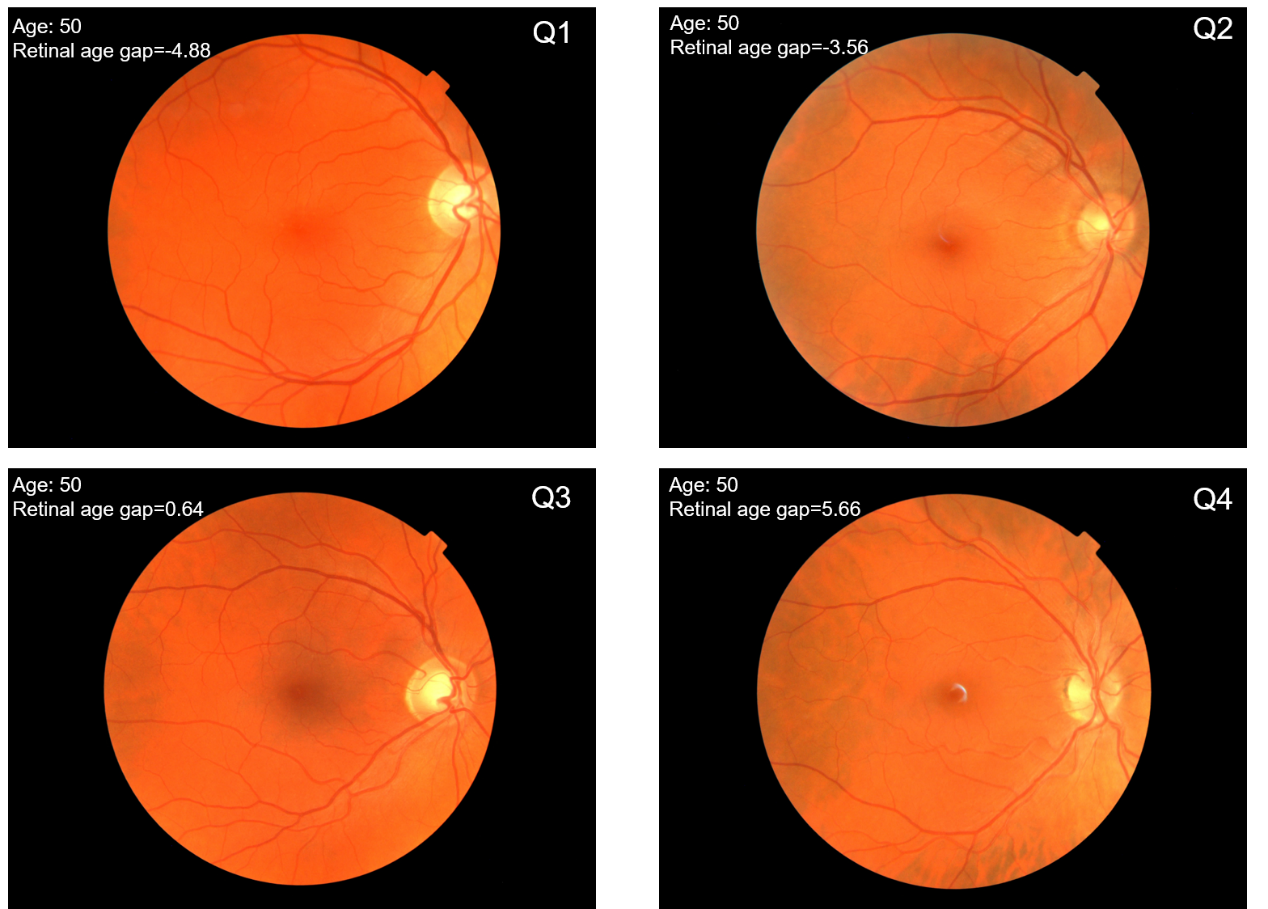

Supplement: Supplementary file 1 — Figure S1. Examples of fundus photographs in different quartiles (Q1–Q4) of retinal age gap in participants aged 50 years. [file JDB-15-237-s001.docx]
